# The role of *WOX1* genes in blade development and beyond

**DOI:** 10.1093/jxb/eraa599

**Published:** 2021-02-27

**Authors:** Michiel Vandenbussche

**Affiliations:** Laboratoire Reproduction et Développement des Plantes, Univ Lyon, ENS de Lyon, UCB Lyon 1, CNRS, INRA, Lyon, France

**Keywords:** Blade development, fruit development, leaflet initiation, medio-lateral polarity, WOX1

## Abstract

This article comments on:

**Wang C, Zhao B, He L, Zhou S, Liu Ye, Zhao W, Guo S, Wang R, Bai Q, Li Y, Wang D, Wu Q, Yang Y, Yan J, Liu Yu, Tadege M, Chen J**. 2021. The WOX family transcriptional regulator SlLAM1 controls compound leaf and floral organ development in *Solanum lycopersicum*. Journal of Experimental Botany **72**, 1822–1835.

***WOX1* genes have been identified in several eudicot species as important regulators of lateral organ development, and were found to control outgrowth of the blade along the mediolateral axis. However, the role of *WOX1* genes in species that have compound leaves remained to be further investigated. Using a reverse genetics approach,** Wang *et al.* (2021 **) analyzed *WOX1* function in tomato and found that *SlLAM1* loss-of-function mutants exhibit defects in leaflet development and leaf complexity. This reveals that *SlLAM1* also plays a critical role in the initiation and growth of leaflets, in addition to its conserved role in blade outgrowth. Interestingly, *SlLAM1* function appears also to be critical for fruit size.**

Final three-dimensional leaf shape depends on the coordinated growth of the leaf primordium along the abaxial/adaxial, proximal–distal, and medio-lateral axes (Fig. 1), and different classes of genes required for the establishment and/or growth along any of these polar axes have been identified (reviewed in Du *et al.*, 2018). Formation of the leaf blade requires growth along the medio-lateral axis, and *WOX1* genes, comprising a distinct subfamily of the Wuschel-like homeobox family (Haecker *et al.*, 2004), were found to be specifically involved in this process in diverse eudicot species (Vandenbussche *et al.*, 2009; Tadege *et al.*, 2011; Nakata *et al.*, 2012; Zhuang *et al.*, 2012; Niu *et al.*, 2018; Wang *et al.*, 2021). Mutations in *WOX1* genes typically result in a reduction of the leaf blade, but without significantly affecting proximal–distal growth, or abaxial/adaxial polarity. During leaf development, *WOX1* genes exhibit a very localized expression pattern restricted to the middle domain region of the leaf primordium, identifying this region as a center that organizes the outgrowth of leaf blades (Nakata *et al.*, 2012). On the other hand, Poaceae such as maize, rice, and barley do not possess *WOX1* genes, and blade outgrowth in these species fully depends on the activity of members of the WOX3 subfamily (Nardmann *et al.*, 2004; Cho *et al.*, 2013; Ishiwata *et al.*, 2013; Yoshikawa *et al.*, 2016).

Fig. 1. **Coordinated three-dimensional leaf growth along developmental axes.**

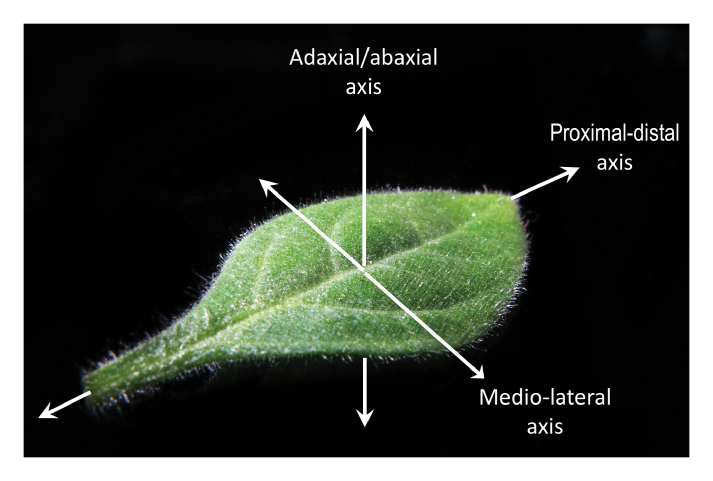

Studies of WOX gene function in various species identify the WOX gene family as particularly dynamic (in part reviewed by Costanzo *et al.*, 2014), and therefore as an interesting target for further comparative (evo-devo) studies. Wang *et al.* (2021) have analyzed the role of *WOX1* in tomato, a species with compound leaf development. Comparison of the results obtained in this study with those of other species further enhances our understanding of the functional diversification of the WOX family and, in the long term, of the evolutionary diversification of the gene regulatory networks in which they are embedded.

## Variations in the phenotypic severity of *WOX1* mutants in diverse species

When comparing *WOX1* mutants in different species, the degree of leaf blade reduction varies significantly. At one end of the spectrum, there is the tobacco *lam1* mutant (Tadege *et al.*, 2011) with virtually no blade tissue remaining, and a similar severe blade reduction was also found by Wang *et al.* in tomato *SlLAM1* mutants (Wang *et al.*, 2021). Arabidopsis is positioned at the other end of the spectrum, as single *AtWOX1* mutants have no obvious phenotype (Vandenbussche *et al.*, 2009). However, leaf blade reduction is observed in *wox1 prs* (wox3) double mutants (Vandenbussche *et al.*, 2009; Nakata *et al.*, 2012), indicating that blade expansion in Arabidopsis is redundantly encoded by members of the WOX1 and PRS (WOX3) subfamilies. Despite this, leaf blade reduction in Arabidopsis *wox1 prs* mutants is clearly less severe compared with the single *WOX1* mutants in tomato and tobacco. This indicates the presence of other factors that can partially compensate for the loss of WOX1/PRS function in Arabidopsis, but apparently not in Solanaceous species such as tobacco and tomato. This suggests that at least part of the molecular network controlling blade development has diverged during evolution of the eudicots. Future understanding of this will require a more integrated view of WOX1 function in the molecular network controlling blade development in diverse species.

Another obvious question that rises from the available comparative data concerns the functional conservation of WOX3 members. The severe blade reduction in *SlLAM1* and *lam1* mutants demonstrates that in contrast to Arabidopsis, *WOX3* genes in tobacco and tomato are not capable of compensating for loss of *WOX1* function, possibly suggesting a different role. The analysis of WOX3 function in these species will be required to validate this hypothesis. Thus far, WOX3 function in dicot species other than Arabidopsis has been analyzed only in *Medicago truncatula* (Niu *et al.*, 2015), showing that loss of function of the *WOX3* ortholog *LFL* (*LOOSE FLOWER*) affects floral development, but not leaf blade outgrowth. In addition, the leaf blade phenotype of *stf lfl* double mutants was found to be similar to that of *stf* [*STENOFOLIA* (*WOX1* ortholog)] single mutants, indicating that *LFL/MtWOX3* has no overlapping function with *STF* in blade development (Niu *et al.*, 2015).

An unexpected result from the study of tomato *WOX1* mutants (Wang *et al.*, 2021) is that *SlLAM1* plays a critical role not only in leaf blade development, but also in the initiation and growth of leaflets in compound leaf development. Earlier studies in other species with compound leaves showed that WOX1 function is required for blade development but does not affect leaflet initiation, as illustrated by the maintenance of trifoliate identity in *stf* (Tadege *et al.*, 2011) and multifoliate identity in *lath* (Zhuang *et al.*, 2012). Tomato and pea/*Medicago* are representatives of the Asterids and Rosids, respectively, which constitute the two largest clades within the core eudicots. These two major clades are thought to have diverged >100 million years ago (Moore *et al.*, 2010). Therefore, differential recruitment of WOX1 function in leaflet initiation might reflect basic differences in the evolutionary changes that led to compound leaf development in distant species. Indeed, a systematic comparison of the molecular players known to regulate compound leaf development in four model organisms (tomato, *Medicago*, pea and cardamine) indicated already that although some mechanisms are clearly conserved, other components of the compound leaf regulatory network may have diverged significantly (reviewed in Bar and Ori, 2015).

## Transcriptional networks regulated by *WOX1* genes in diverse species

In recent years, several genome-wide datasets have become available aimed at the elucidation of the transcriptional network regulated by *WOX1* transcription factors (Tadege *et al.*, 2011; Nakata *et al.*, 2018; Niu *et al.*, 2018), including the study discussed here in tomato (Wang *et al.*, 2021). RNAseq analysis comparing the *slwox1* mutant with the wild type revealed a large number of differentially expressed genes (DEGs), of which the large majority are up-regulated in the *slwox1* mutant background. This is consistent with previous studies and further supports that WOX1 transcription factors act mainly as transcriptional repressors (Lin *et al.*, 2013; Zhang *et al.*, 2014). A further commonality in these studies is the strong enrichment of DEGs belonging to plant hormone signal transduction pathways, of which of particular interest is the auxin pathway. Furthermore, it was recently found in Arabidopsis that MONOPTEROS (MP) [also called AUXIN RESPONSE FACTOR5 (ARF5)] can directly up-regulate *WOX1/PRS* expression while ARF2/3/4 act as negative regulators, and that the adaxial/abaxial distribution of these ARF factors together with auxin collectively defines *WOX1* and *PRS* expression (Guan *et al.*, 2017). This suggests that in Arabidopsis at least part of the leaf developmental defects observed in various *arf* mutants can be attributed to deregulation of *WOX1/PRS* expression. Furthermore, using an inducible Arabidopsis *WOX1* expression system combined with indole-3-acetic scid treatments, Nakata *et al.* (2018) found, among others, that *WOX1* positively regulates *MP* expression and that *WOX1* and auxin additively influenced common downstream target genes. Together, this suggests that WOX1 function and the auxin (signaling) pathway are tightly intertwined through complex feedback loops.

## A bright future for comparative evo-devo studies

Since the beginning of the 1990s, tremendous progress has been made in the understanding of the molecular mechanisms that underlie plant development. For obvious reasons, most advance has been made in Arabidopsis, for which detailed gene regulatory networks have emerged covering a wide variety of developmental processes. We are, however, still very far from understanding how evolutionary variations in these networks have shaped the astonishing morphological diversity in the plant kingdom. Indeed, this requires that a wide range of detailed gene functional studies of key developmental regulators in species with contrasting architecture become available. The recent technological revolutions in next-generation sequencing methods (e.g. RNAseq) and gene functional analysis [clustered regularly interspaced palindromic repeats (CRISPR)/CRISPR-associated protein 9] will strongly accelerate this process and will deliver robust datasets required for comparative studies. The study of *SlLAM1* function by Wang *et al.* is a perfect illustration of this. They analyzed WOX1 function in a different developmental context (compound versus simple leaf development), and characterized the gene regulatory network downstream of tomato SlWOX1 function, allowing comparison with other datasets obtained in different species.
